# Post-stroke depression and the aging brain

**DOI:** 10.1186/2049-9256-1-14

**Published:** 2013-08-23

**Authors:** Gabriel R Cojocaru, Aurel Popa-Wagner, Elena C Stanciulescu, Loredana Babadan, Ana-Maria Buga

**Affiliations:** Department of Functional Sciences, Center of Clinical and Experimental Medicine, University of Medicine and Pharmacy of Craiova, Petru Rares str., no 2, Craiova, 200349 Romania; Faculty of Pharmacy, Chair of Biochemistry, University of Medicine and Pharmacy of Craiova, Craiova, 200349 Romania; Department of Psychiatry, University of Medicine Rostock, Rostock, Germany

**Keywords:** Aging, Stroke, Post stroke depression, Gene profiling

## Abstract

Ageing is associated with changes in the function of various organ systems. Changes in the cardiovascular system affect both directly and indirectly the function in a variety of organs, including the brain, with consequent neurological (motor and sensory performance) and cognitive impairments, as well as leading to the development of various psychiatric diseases. Post-stroke depression (PSD) is among the most frequent neuropsychiatric consequences of cerebral ischemia. This review discusses several animal models used for the study of PSD and summarizes recent findings in the genomic profile of the ageing brain, which are associated with age-related disorders in the elderly. Since stroke and depression are diseases with increased incidence in the elderly, great clinical benefit may especially accrue from deciphering and targeting basic mechanisms underlying PSD. Finally, we discuss the relationship between ageing, circadian rhythmicity and PSD.

## Review

### Background

Depression in stroke survivors is of utmost clinical relevance. It often takes a chronic course and is associated with increased morbidity, mortality and a poorer functional outcome. Despite the fact that a high proportion of stroke patients develop mood symptoms, the pathomechanisms underlying the development of post-stroke depression (PSD) have so far received little attention from the field of neurobiology. Relevant animal models have only sparsely been investigated. This research gap becomes even more regrettable if one considers the growing body of clinical evidence indicating a beneficial effect of antidepressants and especially of selective serotonin reuptake inhibitors (SSRIs), on post-ischemic outcome. Since old age as such is also associated with an enhanced susceptibility to stroke along with a poorer recovery from brain injury, it deserves to be investigated as a key modulatory factor*.* If we cannot prevent stroke, we shall try to alleviate its long-term consequences. In particular, great clinical benefit may accrue from deciphering and targeting basic mechanisms underlying chronic PSD in aged animals. So far, the majority of experimental stroke studies have concentrated heavily on acute stroke outcome, which, after all, represents only a snapshot of a complex sequence of events. This limitation may have majorly contributed to the conspicuous discrepancy between laboratory and clinical findings that has been a recurrent theme in stroke research in recent years (‘translational road block’).

### Post-stroke depression & aging

Age is the most important risk factor for cerebral ischemia and recovery after stroke is significantly influenced by age. A large spectrum of factors, like genetic, epigenetic or environmental factors, contributes to the aging phenotype. One prospective population-based study estimates that the incidence of mental illnesses like anxiety, anhedonia and depression after stroke is about 35% among the stroke survivals and the rate of disabilities and cognitive defficits increasesed with age [1]. Depression after stroke runs a chronic course and is related to increased morbidity and mortality [2–9]. More than that, depression symptoms may even worsen during the chronic phase after stroke [1, 9, 10]. Anxiety is associated with physical disability may contribute to the development of PSD. However, the higher prevalence of symptoms of depression in stroke patients as compared with other patients with similar degree of disability can be a good argument against psychological explanations of PSD [9, 11].

Comorbidities such as hypertension, obesity, diabetes, dyslipidemia and systemic inflammation increase the probability of silent strokes. Microvascular changes and silent strokes in vulnerable regions may lead to the so-called ‘vascular depression’ [12, 13]. Several genes such as the genes encoding angiotensin-converting enzyme (ACE), protein kinase C (PRKCH), apolipoprotein (a) [apo(a)] and lipoprotein(a) [Lp(a)] may play an important role in the ethiology of vascular depression [14–16].

### Animal models of stroke and post-stroke depression: role of aging

To study the biological processes underlying functional recovery after stroke in ageing brain a variety of physiologically complex organisms like rats, mice or nonhuman primates have been used. But, the rat model is by far the most used in stroke research due to the similarities with human brain neurovascular branching and the available behavioural outcome measurements. The most commonly used ischemic stroke models in rodents are: middle cerebral artery occlusion (MCAO) for transient or permanent occlusion and endothelin-1 model for transient occlusion. To study the rehabilitation process after cerebral ischemia is important to choose an appropriate animal model and to optimize this model. Epidemiological studies reveal that human ischemic stroke occurs frequently in late middle age (50-70 years) than at older ages (over 70 years) [17, 18]. Therefore it is highly recommened to use middle aged rats for stroke studies. Consequently, animal studies conducted on aged (18 month-old) rats demonstrated that there was a decline in the ability of aged brain to sustain plasticity-related process and poorer neurological functional recovery after ischemia in older rats than in younger animals [19–25]. Other research studies that used middle-aged rats (12-18-month) showed that more expressed alteration have been found compared with young animals at structural and functional levels [24, 26–29]. Interestingly, there are significant differences in brain response to injury in old subjects compared with young ones. Therefore extrapolating the results from young animals to aged humans could lead to erroneous conclusions.

The aged rodent model offers a useful tool to investigate mechanisms and treatments of ischemic stroke in preclinical studies. The models in aged animals have to be designed to create a reproducible lesion which mimics the human pathophysiological changes, to be minimally invasive, and to allow objective measurement and analysis of tissue damage after cerebral ischemia. In agreement with this concept, previous studies have shown that mortality in post-stroke aged rate is higher compared with young animals, most likely because the lesion appears on a background already altered by senescence itself. On the physiological level, functional and cognitive decline are closely connected to morphological changes of the brain during the aging process.

Imaging techniques, positron emission tomography (PET) or magnetic resonance imaging (MRI), have revealed a significant reduction in the cerebral blood flow (CBF), mostly in the cortex, which may be linked to these morphological changes in the aged brain. Overall, cerebrovascular dysfunction associated with metabolic changes due to senescence increases the vulnerability of brain to ischemic-hypoxic injuries like stroke. Cerebral ischemia occurs frequently in elderly, and increased vulnerability of the aged brain leads to unfavorable recovery of physical and cognitive functions. Although imaging techniques have already been used in numerous studies in animal models of stroke, few groups have applied MRI methods to characterize and monitor the dynamics of ischemic lesions in aged ischemic animals [30–33].

The aged brain displays a higher susceptibility to hypoxia compared with young animals in the acute phase of stroke [27, 32]. On MRI images, aged ischemic rats displayed more severe lesions, which were with similar localizations, but higher incidence and more rapid appearance than in the young rats [30, 31]. With the use of functional magnetic resonance imaging (fMRI) it was demonstrated that patterns of bihemispheric reorganization (increase of the fMRI response in the ipsilateral somatosensory cortex and bilateral thalamic activation) after permanent MCAO in aged rats were the same as in young animals, although the overall time course of recovery in aged rats was more prolonged than that in young rats [32]. Studies using electrophysiological techniques, and in particular electroencephalography (EEG), in ischemic aged animals are mostly lacking. EEG has been used as a tool for verifying the success of the occlusion [30], for identifying the effect of hypothermia on neuronal functions [34].

### Animal models of depression

Modelling psychiatric conditions like depression after stroke in animal models is not trivial. The psychological evaluation by clinicians is not available in animal models and most of these models are validated only by behavioural observation or by behavioural changes in response to treatment. Therefore instead of trying to fully replicate all the human symptoms of depression, we shall try to uncover the underlying signalling pathways in animal models of mood disorders that strongly meet the validating criteria including strong endophenotype similarities, comparable etiology and the same treatment [35–37]. To this end, various behavioral tests have been proposed to investigate some of the central aspects of human-like depression in rodents. For example, the forced swim test in which rodends are exposed to water stress and are forced to swim [9, 38] or the tail suspension test (animals are suspended horizontaly by tail for a short period of time) [39, 40] are commonly used as behavioral paradigms that quantify behavioral changes in a stressful situation (behavioral despair). These tests measure the immobility of depressed animals in despair situation and have been pharmacologically validated using antidepressant drugs that are already in human use [41, 42].

Anhedonia (the loss of interest) is an important symptom of depression that can be measured in rodents by a decrease in sucrose consumption. Rodents normally prefer sweet fluids like glucose or sucrose instead of water. Quantifying consumption of sucrose is the most used endpoint for assessing motivation and affective state in rodends after repeated chronic stress exposure. Also, this test can quantify reversal of this effects after antidepressive drugs administration [9, 43–45]. Some studies report decreased sucrose consumption at 2 weeks after transient focal ischemia in mice, suggesting a hedonic deficit in MCAO animals [40, 44–46].

Exposure to unpredictable chronic mild stress (CMS) associated with isolation of animals after ischemia is another way to study experimental PSD. It has been shown that after cerebral ischemia, animals show decreased locomotor activity in the open field test and decreased sucrose consumption when exposed to CMS paradigm for 18 days after surgery [9].

### Biology of post-stroke depression: role of ageing

The high incidence rates of stroke patients that develop mood symptoms (between 20-50%) justify the effort of researchers to go further into the neurobiological mechanisms of disease [47–49]. Many studies suggest that PSD is a consequence of brain lesions that are associated with disruptions in synaptic transmission, changes in signalling pathways and increased biological vulnerability of the post-stroke aged brain [50–53]. Some other studies reported that PSD is a consequence of specific brain lesions and differences in the incidence of depression between different brain areas have been reported [54, 55]. In this context, left hemispheric cortical stroke, mainly frontal lesions has been reported to be linked with an increased risk for depression. However, there are still controversial points of view regarding the relationship between the area of the brain affected by stroke and incidence of PSD.

On the other hand, the prevalence of the memory cognitive impairment like dementia or depression is higher in elderly after stroke. One question is that if cerebral ischemia causes secondary degenerative changes in the brain or that ongoing degenerative changes will be simply aggravated by stroke. From a psychological perspective, the severity of PSD is determined not only by individual differences in emotional reactions to disease (e.g. negative attitude) but also, by the severity of physical and cognitive impairment and by the absence of familial and social support [56].

Many studies suggest that post-stroke vulnerability of the brain can induce PSD and PSD is associated with reduced recovery after stroke in stroke survival patients. However, until now there is no clear evidence to support the etiological mechanisms of PSD, which seems to be a multifactorial disease of the ageing brain.

One important issue is how to distinguish the depressive symptoms in patients in the early stages after stroke from cognitive impairments due to neurodegeneration prior to stroke and the ageing process itself. Some longitudinal studies on post stroke patients showed that chronic PSD is highly predictable if post stroke patients are experiencing depression symptoms between 6 month and 1 year after brain injury [57, 58].

Most of these studies analyzed the risk of post-stroke depression in relatively young people’s that have a job and are not living alone. Also, in these studies, patients with language problems like aphasia or neurodegenerative disease like dementia where excluded. However, since stroke occur frequently in people over 65, studies on older patients with stroke and other age-related comorbidities should be more relevant than studies on young people. In this light, multi-therapeutic approach of PSD in the recovery phase that include genetic, social and psychological aspects have the greatest potential for improving post-stroke recovery and the quality of life in elderly post-stroke survivors.

### Neurogenesis, cognitive decline & post-stroke depression

Age-related cognitive decline is often associated with decreassed hippocampal neurogenesis and depression, but relatively little is known about the biological significance of neurogenesis in the ageing mammalian brain for the development of depression. Two major hypotheses have driven most of the studies on hippocampal neurogenesis, namely (i) it plays a pivotal role in hippocampus-dependent learning and memory [59, 60] and, (ii) it protects against anxiety and depression [61, 62]. However, mechanisms underlying the precise role of neurogenesis remains controversial. For example, genetic ablation of the cell cycle regulatory protein cyclin D2 that results in virtual absence of newly born neurons in the adult brain does not lead, surprisingly, to appreciable learning and memory deficits [63–65]. Similarly, the involvement of hippocampal neurogenesis in depression and in the efficacy of antidepressive treatments is also not fully understood.

One possible molecular mechanism underlying age-related depression and decreased neurogenesis can be due to an increased level of the dickkopf 1 homolog - Xenopus laevis (Dkk1), that decreases Wnt signaling pathways and has been associated with a decline in hippocampal neurogenesis [66].

Other mechanisms that can be involved in neurorecovery are related to neurotrophin signaling pathway. Neurotrophins are important players in early neuronal gene response to injuries. The neutrophin-signaling pathway activates extracellular-signal-regulated kinases (ERK) pathway and nuclear transcription. Meier and colleagues demonstrated that hippocampal neuronal culture treated with brain derived neurotrophic factor (BDNF) promotes axonal guidance, modulate the synaptic function, stimulate neurite branching and is antagonized by Ephrin (Eph) signaling [67, 68]. Also, decreased levels of BDNF, a key factor in the regulation of hippocampal neurogenesis, seems to be associated with depression and neurodegenerative disorders, but the mechanisms underlying this association are still unknown [69]. Finally, Cui and colleagues reported that the combination therapy, simvastatin with human umbilical blood cells, increased endogenous neurogenesis and cell plasticity in the ischemic area via BDNF/TrkB signaling pathway [70].

Even less is known about the relationship between PSD and neurogenesis in the elderly. The level of hippocampal neurogenesis has been shown to decrease steadily with aging [71]. Since aged animals might be both more prone to develop a depressive phenotype [72] and the aged brain is more sensitive to the deleterious effects of ischemia [27, 73], one could expect more severe PSD symptoms in aged animals. Such an experimental model of PSD, taking into account these influences of aging, should be highly clinically relevant.

Depressive behavior in ischemic rats was accompanied by reduced ischemia-evoked hippocampal neurogenesis and this effect was reversed by citalopram administration [9]. Using pharmacological interventions, the involvement of serotonergic neurotransmission was then further corroborated [74, 75]. One study in non-human primates, proved that the repeated separation stress is associated with depression-like behavior (anhedonia) and reduced hippocampal neurogenesis [76]. Also, recovery from stroke was shown to be associated with growth factor-induced neurogenesis in SVZ as well as exercise-induced neurogenesis in SGZ [77, 78]. Similarly, therapy with granulocyte colony stimulating factor (G-CSF) enhanced neurogenesis, improved working memory in the radial-arm maze test and in consequence the survival capacity and functional outcome after stroke [27]. However, these findings need further confirmation along with a clear demonstration of functional significance in human diseases. We should take into account that other age-associated comorbidities like hypertension or obesity can negatively affect the hippocampal functions.

### Genome profiling of mood disorders in the elderly

Transcriptional profiling is a usefull tool to identify genetic pathways associated with mood symptoms in the elderly. Most studies reporting the use of gene expression profiling to investigate rodent models of depression focused on stress models and did not supply direct evidence for a specific genomic signature in PSD depression. Kang and collegues identified some synaptic-function-related genes that are connected with decreased in number and function of synapse in a rat model of major depression. These genes included: calmodulin 2 (*Calm2*), synapsin 1 (*Syn1*), tubulin beta 4 (*Tubb4*) a member of ras-related protein Rab-4B (*Rab4b*). Also, increased expression of the transcriptional repressor erythroid transcription factor/ GATA-binding factor 1 (GATA1) is responsible for downregulation of these synaptic-function-related genes [79].

In another study, genes related to human major depression like serotonin receptor 2a gene (*Htr2a*), neurotrophic tyrosine kinase receptor type 2 and 3 genes (*Ntrk2* and *Ntrk3*), corticotropin releasing hormone receptor 1 (*Crhr1*) and corticotropin releasing hormone (*Crh*) were differentially expressed in three animal models of depression: acute treatment with reserpine, olfactory bulbectomy and chronic treatment with corticosterone [9]. In addition, two new genes, complement component 3 and fatty acid-binding protein 7, have recently been described [80, 81]. Similarly, then polymorphism of 5- hydroxytryptamine 2a receptor (Htr2a), a postsynaptic target for serotonin signaling, has been implicated in neuropsychiatric disorders [82]. In addition, increased functional activity of the amygdala in response to negative stimuli appears to be a mood-congruent phenomenon that is likely moderated by the 5-HT transporter gene (*Slc6a4*) promoter polymorphism (*5-Httlpr*) [9]. Lohon and colleagues showed significant gene-gene interaction between *Slc6a4* and *5-Httlpr*/rs25531 in general anxiety disorder [83].

An oligodendrocyte/myelin-associated genes, 2’,3’-cyclic nucleotide 3’-phosphodiesterase (CNP*)* was identified to be associated with catatonia-depression syndrom in the elderly. Using aged heterozygous null mutant mice model of spontaneous catatonia, Hagemeyer and collegues showed that the reduced expression of CNP is accelerated by aging and is associated with neurodegenerative changes in the elderly [84].

### Gene expression & mood disorders in elderly

In previous studies we have identified a number of genes that are involved in neuropathic syndrome and PSD signaling pathways in aged brain (e.g. 5- hydroxytryptamine 2a receptor - *Htr2b*, prepronociceptin *- Pnoc*). These genes could be pharmacological targets in a multimodal therapy of stroke and stroke related diseases [16].

Using fosB-Null mice, Yutsudo and colleagues reported impaired neurogenesis and depressive behavior in fosB-Null mice [85]. Intriguingly, FBJ murine osteosarcoma viral oncogene homolog B *(fosB)* expression has been associated with stem cell and neural progenitor cells proliferation after cerebral ischemia in mammalian central nervous system [86, 87]. These studies suggest the genomic signature is crucial for the evolution of disease, but is the “genomic reprogramming” a future powerful tool that can be exploited to improve the neurorecovery after stroke? Some studies identified the ciliary neurotrophic factor (Cntf) receptor as a key molecular factor that can inhibit neurogenesis in the type B stem cells, but the mechanism is still unknown [88, 89]. Cntf is expressed only in central nervous system where modulates the normal neurogenesis. Stimulation of this factor can be a novel pharmaceutical strategy for neurogenesis-dependent diseases like stroke and PSD [89]. Table 1 summarize all the specific genes involved in the ethiology of depression and post-stroke depression.

**Table 1:** **Specific genes involved in the ethiology of depression and post**-**stroke depression**.

Gene symbol	Description	Gene Function	Gene expression	Disease	Human/animal data	Ref.
***Depression***						
GSK- β	Synthase-kinase-3β	Central regulator of circadian rhythms	Up	Depression	Transgenic mice	[90]
CLOCK	Circadian Locomotor Output Cycles Kaput	Central regulator of circadian rhythms	SNP	DepressionBipolar disorders	Transgenic mice	[91]
ARNTL(BMAL1)	Aryl hydrocarbon receptor nuclear translocator-like	PER1 activator	SNP	Sleep disorders	Human sample,	[92, 93]
NPAS2	Neuronal PAS domain protein 2	Part of a molecular clock	SNP	Mood disorders	Human sample,	[92, 94]
***Synapse-related genes in depression***						
CALM2	Calmodulin 2	Cytokinesis regulator	Down	Depression	Animal model	[89]
SYN1	Synuclein1	Synaptogenesis and neurotransmitter release	Down	Depression	Animal model	[89, 95]
***Depression in the elderly***						
PER2	Period circadian clock 2	Central regulator of circadian rhythms	SNP	Sleep disorders Ageing brain	Human sample Animal model of ageing	[92, 93, 96]
			Up			
PER3	Period circadian clock 3	Central regulator of circadian rhythms	SNP	Sleep disorders, Aged brain	Human sample	[93, 97–99]
5-HTTLPR	Serotonin transporter promotor	Serotonin transporter	SNP	Depression in the elderly	Human sample	[100]
TUBB4	Tubulin, Beta 4A Class IVa	Constituent of microtubules	Down	Depression Ageing	Animal model	[89, 101]
***Depression and recovery after stroke***						
BDNF	Human brain-derived neurotrophic factor (BDNF)	Growth factor in the brain	SNP	Depression Recovery after injury	Human sample, Animal model	[102, 103]
SLC6A4	Solute Carrier Family 6 Member 4	Membrane protein transporter of serotonin	SNP	Depression Stroke recovery	Human sample	[9, 83]
GATA1	GATA Binding Protein 1	Transcription factors	Upregulation	Depression Stroke recovery	Animal model	[104]
HTR2B	5-Hydroxytryptamine (Serotonin) Receptor 2B	Serotonin receptor	Upregulation	Poststroke depression in elderly	Animal model	[16, 34]
PNOC	Prepronociceptin	Opioid receptor	Downregulation	Poststroke depression in elderly	Animal model	[16, 34]

### Mood disorders, circadian rhythmicity and aging

Disturbances in the circadian rhythm may have dramatic effects on our health. Changes in biological rhythm disturbances precede and parallel the occurrence of mood episodes of illness and have been proposed to play a pathogenetic role in major depression and mania [105–110]. The controlled administration of stimuli that can directly act on the clock, namely light and manipulations of the sleep-wake rhythm, has established high efficacy in the treatment of mood episodes, also in drug resistant patients. Effects of Total Sleep Deprivation and Light Therapy on the phase of biological rhythms could be part of its mechanism of action.

To understand the therapeutic action of these mood stabilizing drugs as well as antidepressants, investigators have recently begun to examine their effects on intracellular signaling pathways that regulate clock gene expression. Yang and collegues [111] utilised an ex vivo approach to examine circadian rhythms in clock gene expression profiles in fibroblasts either obtained from bipolar disorder patients or healthy controls, and report that gene encoding for a basic helix-loop-PAS domain (bHLH-PAS domain) transcription factor (BMAL1), period circadian protein homolog 1 (PER1), period circadian protein homolog 1 (PER2), nuclear receptor subfamily 1, group D, member 1 (REV-ERB- α) and the clock controlled gene, D Site Of Albumin Promoter (Albumin D-Box) Binding Protein (DBP), all tended towards reduced amplitudes of circadian oscillation in bipolar disorder.

Assessing the impact of agomelatine on depressed bipolar patients [112], while measuring their circadian rhythms, may therefore help to further precise if it is through the restoration of circadian rhythms that agomelatine get treatment reponse (assessed by actimetry), and help to pinpoint which genes expression are being specifically modified (from fibroblasts). Diurnal rodents to decipher the relationship between circadian rhythms and depression. One of the major obstacles in the developent of appropriate models for circadian rhythm disturbances-related psychiatric diseases may arise from the fact that the standard animals used in neuropsychiatric research are nocturnal rodents. Despite of the extraordinary advancement in our understanding of the circadian clock mechanism, it is still unclear how are the temporal signals from the clock translated into activity patterns, and how do they differ in diurnal and nocturnal mammals.

Nevertheless, it is clear that some fundamental differences exist between nocturnal and diurnal mammals which may be crucial for the study of circadian rhythms related diseases [113–115]. For example much like humans, diurnal species are active when melatonin levels are low, while nocturnal mammals are active when melatonin levels are high. Another important component of the circadian system is the masking effect of light. Specifically, light increases activity in diurnal mammals (positive masking) and suppresses it in nocturnal ones (negative masking), while darkness acts in the opposite ways [116, 117]. Therefore we suggest that using diurnal animals to decipher the molecular mechanisms underyling the relationship between circadian rhythms and affective behavior [118].

Circadian rhythms display an unregular pattern with aging manifested by alteration of sleep quality and cognitive performance [119, 120]. Hermannn and Bassetti [121] showed that the alterations of the sleep-wake cycle like hypersomnia or excessive daytime sleepiness occur in 10%-50% of all stroke cases and are associated with negative long-therm clinical outcome. Also, Ramar and Surani [122] showed that the circadian rhythm disorders could increase the risk of stroke. But, if disturbances in the circadian rhythm are a risk factor or a consequence of ischemic stroke in the elderly remains to be clarified.

Some studies showed that one mechanism that contributes to increased risk of depression is the decrease in the synthesis of N-acetylserotonin with ageing [123]. Since N-acetylserotonin activates TrkB signaling pathway in a circadian fashion (higher in the night and lower during the day) via TrkB receptor, and has antidepressant effects [124] it has been hypothesized that disturbances in the circadian rhythms may cause psychiatric disorders. For example, Bunney and colleague showed that an altered circadian function and altered expression of the central circadian clock genes, BMAL1/CLOCK (Npas2) in mood disorders [125]. Also, Circadian Locomotor Output Cycles Kaput (CLOCK) genes are strongly involved in the circadian rhythm and these are closely related with external factors [126]. Therefore dysfunctions of circadian time regulatory mechanisms in the aged brain may underlie the etiology of PSD in the elderly. The effect of circadian rhythm on PSD outcome in the elderly is still an unexplored field.

### Therapy of post-stroke depression

Norepinephrine (NE), serotonin (5-HT), and dopamine (DA) overlap in the brain and all three transmitters are implicated in the symptoms of depression Depressive symptoms may result from dysfunction of any or all of the monoamine neurotransmitter systems. The effects of NE, 5-HT and DA overlap in the brain and all three transmitters are implicated in the symptoms of depression. Because these monoamine transporters (MATs) are important regulators of the extracellular neurotransmitter concentration, mouse gene knockouts of serotonin transporter (SERT), the noradrenaline transporter (NAT) and also the dopamine transporter (DAT) located in the plasma membrane of corresponding neurons provide interesting models for possible effects of chronic antidepressant treatments. Inhibition of neurotransmitter reuptake by drugs acting at SERT, NET and/or DAT can produce antidepressant effects [127, 128].

The mechanism of PSD was suggested to involve multiple pathways, like immune activation, hypoxia, apoptosis and necrosis of neuronal or glial cells or hyperactivation of the hypothalamic-pituitary-adrenal axis. Many studies reported different therapeutic strategies designed to improve the PSD outcome. Of these, cortisol-lowering therapies and increases of neurotropic factors like BDNF were reported to be novel possible therapeutic strategy for PSD [129].

In addition, a growing body of evidence indicate a beneficial effect of antidepressants and especially of SSRIs on postischemic outcome [9]. Antidepressants may also exert direct actions on the brain, providing neuroprotection and promoting brain plasticity and neurogenesis.

Antidepressants treatment initiated soon after stroke in non-depressed post-stroke patients may prevent the later PSD but the time window of treatment remains to be optimized [130]. A number of studies have also reported beneficial effects of antidepressant pharmacotherapy on long-term functional outcome after stroke including activities of daily living as well as cognitive functioning [9, 131–135]. Other in vivo and in vitro studies have shown that fluoxetine and paroxetine which are the most commonly prescribed antidepressants, prevented degeneration of nigrostriatal dopaminergic neurons. These drugs reversed the hypoactivation found in the primary motor cortex of patients [136] and the increased activation was correlated with improved performance after drug intake and repression of proinflammatory markers [9]. These results remain, however, to be validated in large clinical trials of stroke patients.

## Conclusions

In conclusion, depression is the most frequent neuropsychiatric disease of brain ischemia, affecting up to 35% of all such patients. PSD is associated with negative outcome of functional recovery, cognition and social reintegration of stroke patients. During de past decade, significant efforts have been made to establish an efficient treatment of PSD in the elderly. So far, preclinical and translational research on PSD is largely lacking. The implementation and characterization of suitable animal models is clearly a major prerequisite for deeper insights into the biological basis of post-stroke mood disturbances and may also pave the way for the discovery of novel therapeutic targets. Nevertheless it is unlikely that monotherapies will provide a cure for PSD. Rather multitherapeutic strategies should be at the focus of future clinical trials conducted on PSD and mood disorders patients without cerebral ischemia that show the same clinical profile. In this light, future research is needed to identify the molecular mechanism of disease and to establish the pathways that are modulated by antidepressant drugs leading to a better cognitive recovery in the elderly patients.
